# A New Myohaptic Instrument to Assess Wrist Motion Dynamically

**DOI:** 10.3390/s100403180

**Published:** 2010-04-01

**Authors:** Mario Manto, Niels Van Den Braber, Giuliana Grimaldi, Piet Lammertse

**Affiliations:** 1 FNRS, Neurologie ULB-Erasme, 808 Route de Lennik, 1070 Bruxelles, Belgium; 2 Moog FCS, 2150 Ad Nieuw-Vennep, The Netherlands; E-Mails: nvandenbraber@moog.com (N.V.D.B.); plammertse@moog.com (P.L.); 3 Neurologie, ULB Erasme, 808 Route de Lennik, 1070 Bruxelles, Belgium; E-Mail: giulanagrim@yahoo.it (G.G.)

**Keywords:** movement, sensor, myohaptic, damping, ataxia

## Abstract

The pathophysiological assessment of joint properties and voluntary motion in neurological patients remains a challenge. This is typically the case in cerebellar patients, who exhibit dysmetric movements due to the dysfunction of cerebellar circuitry. Several tools have been developed, but so far most of these tools have remained confined to laboratories, with a lack of standardization. We report on a new device which combines the use of electromyographic (EMG) sensors with haptic technology for the dynamic investigation of wrist properties. The instrument is composed of a drivetrain, a haptic controller and a signal acquisition unit. Angular accuracy is 0.00611 rad, nominal torque is 6 N·m, maximal rotation velocity is 34.907 rad/sec, with a range of motion of −1.0472 to +1.0472 rad. The inertia of the motor and handgrip is 0.004 kg·m^2^. This is the first standardized myohaptic instrument allowing the dynamic characterization of wrist properties, including under the condition of artificial damping. We show that cerebellar patients are unable to adapt EMG activities when faced with an increase in damping while performing fast reversal movements. The instrument allows the extraction of an electrophysiological signature of a cerebellar deficit.

## 1. Introduction

Fast single-joint monodirectional movements are associated with a triphasic pattern of electromyographic (EMG) activity: a first burst in the agonist muscle (providing the launching torque) is followed by a second burst in the antagonist muscle (providing the braking torque), followed by a second burst in the agonist muscle (to bring the limb accurately to the target) [1,2]. In 1998, Gottlieb described the main kinematic and EMG features of reversal movements in healthy subjects (a reversal movement is a movement towards a fixed target followed immediately by a return to the initial position). Reversal movements are balanced in shape, and the agonist EMG activity is composed of 2 bursts which are clearly separated [3]. During a fast voluntary movement, muscle damping is typically asymmetrical, predominant in the direction of muscle shortening [4]. For hand kinematics in the physiological range of motion, the damping compensation signal is a crucial element for kinetic encoding by the motor cortex, which generates the corticomotoneuronal discharges towards the end effectors [4]. The structures in the central nervous system (CNS) regulating the damping compensation signal have not been identified so far. Although it is widely accepted that the cerebellum regulates the planning and the execution of voluntary movements [5], the contribution of the cerebellar pathways in the damping compensation signal has remained elusive.

Cerebellar patients are typically clumsy, performing movements which are dysmetric. Hypermetria (overshoot of the target) is the most common form of dysmetria in cerebellar patients [6]. Hypermetria is associated with the following electromyographic (EMG) deficits: a delayed onset latency of the antagonist EMG activity, a decrease rate of rise in the antagonist EMG activity, an inability to increase the intensity of the agonist and antagonist EMG activities when the inertia of the moving limb is artificially increased [7–10]. The second form of dysmetria is the undershoot (hypometria), which is observed in hypokinetic syndromes, in cerebellar disorders and in diseases combining cerebellar and extra-pyramidal symptoms. Hypometria can also occur as a result of an aberrant recovery from a cerebellar lesion [11].

In assessing metrics of motion in neurological patients, the selection of the instrumentation is critical [12]. Indeed, assessment of joint properties and voluntary motion in neurological patients remains a challenge in daily practice. Several tools have been developed these last decades, but so far most of them have remained confined to laboratories, mainly due to a lack of standardization, the difficulty to implement them in a clinical environment or the absence of clinically-relevant information provided by the instrumentation. We report on a new myohaptic device (the terminology “myohaptic” designates the use of myoelectric signals in combination with a haptic interface in a sensor fusion approach, with the possibility to control the motor directly from the muscle discharges recorded with needle or surface EMG), called wristalyzer, which allows assessment of the metrics of motion under various damping conditions. We show an inability of cerebellar patients to tune appropriately the intensities of muscle discharges in a task requiring a sequential activation of muscles. As compared to previous works [6–12], the present paper extends the application of the wristalyzer to a group of cerebellar patients and shows for the first time (1) how kinematic and electromyographic data correlate with clinical observations, and (2) the assessment of stretch reflexes in cerebellar disorders.

## 2. Methods

**Figure 1.**
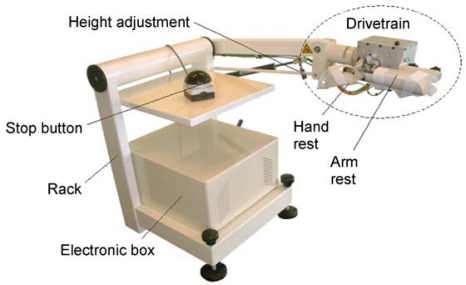
Experimental device (wristalyzer). The mechatronic myohaptic unit includes a moving unit and its controller. The system comprises a direct drive brushless motor with a high resolution encoder. Sampling rate is 2,048 Hz per channel. A high degree of stability on the floor is ensured by adjustable screws.

Studies were performed following approval of the institutional ethical committee of the Free University of Brussels. Subjects signed a written consent to participate in the study. We built a device which allows the investigation of motion of the wrist under several mechanical conditions with a high degree of accuracy, and which takes into account the ergonomy of the upper limbs (Figure 1).

The device is based upon haptic technology, allowing instantaneous regulation of the mechanical characteristics of the manipulandum. In order to reduce the inertia of the manipulandum and decrease the dynamics perceived by the subject, a haptic controller is used. This allows modifications of the mechanical features of the manipulandum in a rotational inertia-spring-damper system. The haptic inner loop runs on an embedded haptic server computer running a real time operating system on a 2,048 Hz interrupt generated by a hardware clock. The wristalyzer controller is commanded from a host computer with a Ethernet card (100 Mbps). The wristalyzer is equipped with a safety button and its range of motion is constrained from −1 to +1 rad. The rotational inertia of the motor and handgrip is 0.004 kg m^2^ (it can be configured *via* a web interface). Friction is compensated *via* software. Damping is controlled by multiplying a damping coefficient (*mDampCoef*) to the velocity of the end effector (*mEffectVel*). The result is introduced in the Torque command of the device:

$$\tau_{\mathrm{Output}}=\tau_{\mathrm{TorqueCommand}}-[\mathrm{mDampCoef}\times \mathrm{mEffectVel}]$$

Movements were studied in the basal condition (free mode) and with addition of damping either at 0.1 N·m·s/rad or 0.2 N·m·s/rad. For comparison, damping of a completely relaxed hand is 0.02 to 0.03 N·m·s/rad [13].

All of our subjects were right-handed and we investigated their right hand in all cases. The control group was composed of eight healthy subjects (three women) with a mean age (±SD) of 34.8 ± 10.2 years. We included eight cerebellar patients exhibiting dysmetria in upper limbs (six men, two women; mean age ± SD: 56.2 ± 17.7 years). All the patients had a complaint of clumsiness during performance of voluntary movements. Our patients were scored using the AS20 ataxia rating scale, a clinical scale which has been designed for cerebellar disorders and which is based upon the routine neurological examination [14]. Ataxia scores ranged from 5 to 17, where a score of 20 corresponds to the most severe ataxia. Brain MRI showed a cerebellar involvement in all the patients. A diffuse cerebellar atrophy was found in six patients (mean age ± SD: 50.5 ± 16.1 years; four cases with sporadic cerebellar degeneration and two cases with a genetic ataxia, see also [15]) and two patients exhibited a cerebellar stroke. A gluten ataxia was specifically looked for [16].

Subjects were comfortably seated, with the shoulder relaxed and the upper arm perpendicular to the forearm. The hand and forearm were affixed with straps. The wrist joint was carefully aligned with the motor axis. Movements were performed in the horizontal plane. Subjects performed sets of fast pointing movements (FPM) and fast reversal movements (FRM) over three distances (targets: 0.2, 0.3 and 0.4 rad). The targets were horizontal lines displayed on the screen of a computer placed in front of the subject. The position as measured by the wrist angular measurement device was relayed to the screen as a second line that needed to be aligned with the target line. The origin (0 rad) was defined as the neutral position of the hand. For FPM, subjects were instructed to flex the wrist quickly towards the aimed target. Speed and accuracy were stressed (“you have to perform the movement as quickly and as accurately as possible towards the target displayed on the screen”). For FRM, subjects were instructed to flex the wrist quickly and accurately towards the first target (located at 0.2, 0.3 or 0.4 rad), and to come back immediately to the starting position at 0 rad (“you have to perform the movement as quickly and as accurately as possible towards the first target, and to get back immediately to the initial position as fast and as accurately as possible”). Each subject practiced three to four trials before recordings of FPM, and practiced three to four trials before recordings of FRM. Subjects performed series of 10 fast movements. Each movement started after a “go” signal. We used the following order: 10 fast flexions for each angle (0.2, 0.3 and 0.4 rad from the initial position), followed by 10 fast reversal movements for each angle. For each set of recordings, we studied movements in the basal condition and following addition of artificial damping (0.1 or 0.2 N m s/rad). Therefore, we analysed 30 FPM and 30 FRM in each of the three damping states (60 × 3 = 180 movements per subject). We recorded the surface EMG activities of the flexor carpi radialis (FCR) and extensor carpi radialis (ECR) muscles. Surface EMG activities were amplified by a factor of 1,000, and full-wave rectified (filter settings: 20 to 500 Hz; Delsys surface electrodes, USA; electrodes fixed on the skin with tape). We averaged each set of 10 movements, both for wrist angle data and EMG data. Individual records were aligned to the onset of the agonist EMG burst according to a method described earlier [3]. We did not encounter difficulties to align traces using this method. The averaging process allows a better estimation of the electrical activity generated by the muscle. In addition, averaging has a smoothing effect on the EMG pattern. The averaging procedure not only smoothes the movement data, but allows also a better quantification of the metrics of motion in cerebellar patients.

Calibration of surface EMG activities is critical to compare intensities of agonist and antagonist EMG activities within subjects and across subjects [9]. To this aim, we developed a novel calibration method adapted to the myohaptic device. We assessed the maximal contraction in an isotonic task (MIC, maximal isotonic contraction; the motor is opposing a controlled force). Subjects were asked to perform a maximal wrist flexion (10 trials) and a maximal wrist extension (10 trials) against a torque of 20 Nm controlled by the computer. Corresponding EMG activities were rectified and averaged (EMG activities of the FCR for the flexions, EMG activities of the ECR for the extensions). The calibration area was defined as the integrated area below the averaged EMG trace (traces are first rectified before averaging) corresponding to a torque value from 0 up to 6 Nm. The reliability of this procedure was assessed in four healthy subjects during three successive sessions. The variability of the procedure was lower than 2.9%. For FPM, the following parameters were computed:

- Movement amplitudes
- The integral of the agonist EMG activity (FCR) from onset to peak velocity (acceleration phase, Q_ACC_): an index of the launching EMG activity (activity in the agonist prime mover generating the launching torque)
- The integral of the antagonist EMG activity (ECR) from onset to the second zero-crossing of the acceleration signal (Q_DEC_): an index of the braking EMG activity for the antagonist muscle
- The onset latency of the antagonist EMG activity (Lat_Anta,1_) [3,7,8].

For FRM, we assessed movement amplitudes and the onset latency of the antagonist EMG activity (Lat_Anta,1_; ECR muscle). In order to estimate the adaptation of the braking impulse for the ECR muscle (braking of the first phase of movement from starting position to the first target), we also computed the integrals over the first 80 msec for the antagonist burst in this muscle (Q_80,ANTA_) (an interval of 75 msec has been used in other studies see [3]).

In the subgroup of 6 patients with predominant involvement of the cerebellar cortex, we also assessed the effects of rapid wrist extension movements on the EMG activities of the FCR muscle, in order to evaluate the effects of wrist extensions upon short-latency stretch responses (SLSR) and long-latency stretch-responses (LLSR). Indeed, previous studies have shown that disorders of the cerebellar cortex are associated with impaired tuning of the magnitudes of late components of stretch reflexes [17]. EMG traces were rectified and averaged for 45 trials. Extensions were applied randomly every 5 to 10 seconds. We computed the ratios of the maximal amplitudes of LLSR divided by SLSR (ratios LLSR/SLSR, expressed in arbitrary units). Extensions were imposed *via* a rapid extension of the wrist joint (4.7 rad/sec). The wrist joint was in a neutral position at the onset of the stretch as previously described (see [18]) and subjects were asked to slightly activate their FCR muscle throughout the procedure, using a visual feedback of the EMG activity.

### Stastistical Analysis

Statistical analysis was performed using Sigma Stat (Jandel Scientific, Germany). Polynomial fit (linear, quadratic) and exponential fitting were tested to assess the following relationships: correlation between AS20 ataxia score and movement amplitudes, correlation between AS20 ataxia score and onset latency of the antagonist EMG activity. Best results were achieved with the linear fitting, which was thus selected. The relationship between onset latency of antagonist EMG activity and the ratios of LLSR/SLSR was fitted using an exponential rise to maximum with two parameters:

f=a×(1−exp (−b·x))

For both FPM and FRM, the analysis of variance was used to assess the damping effect in each group and to compare the effects of addition of artificial damping in the two groups (group by damping interaction). A Bonferroni test was subsequently applied for pairwise multiple comparisons. The Mann-Whitney rank sum test was used to compare the ratios LLSR/SLSR in the two groups.

## 3. Results

**Figure 2.**
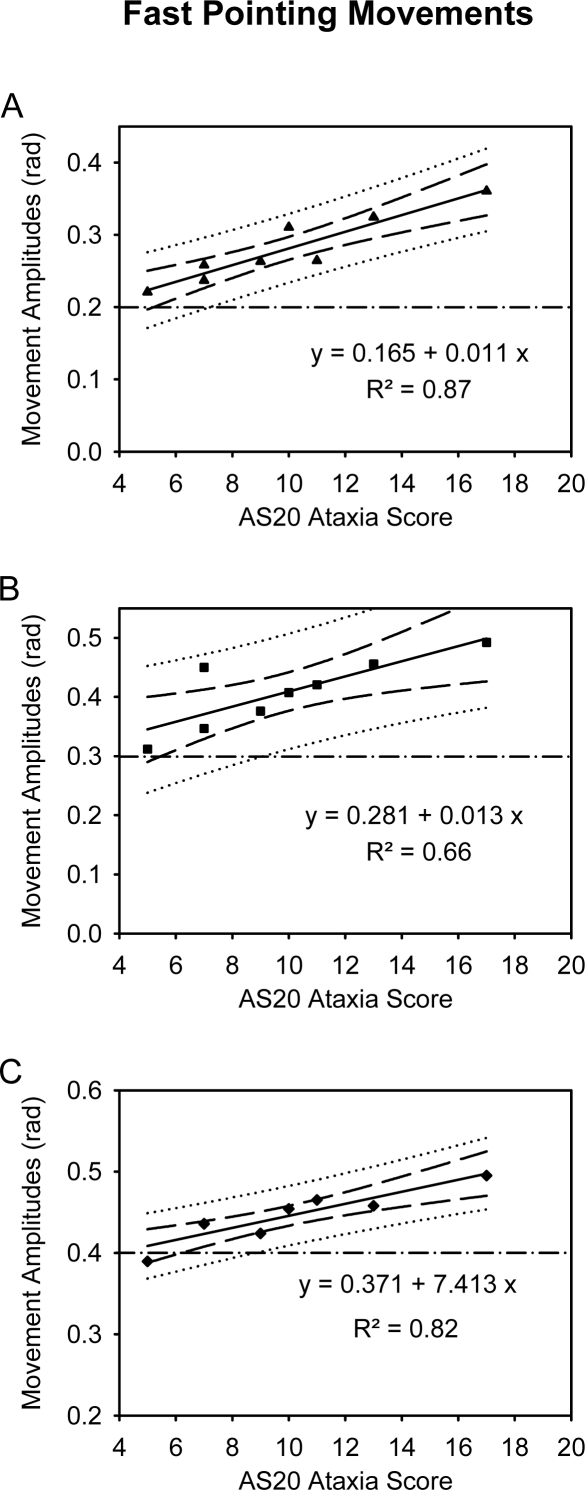
Correlation of the ataxia rating score with the mean movement amplitude in the cerebellar patients during performance of fast pointing movements (FPM) in the basal mechanical condition. Aimed target located at 0.2, 0.3 and 0.4 rad from the starting position in A, B and C, respectively. Long dash: 95% confidence intervals; dotted lines: 95% prediction intervals.

We found a linear correlation between the AS20 Ataxia score and the hypermetria associated with the execution of FPM during the basal mechanical state (Figure 2; p < 0.001, p = 0.014 and p = 0.002, respectively for an aimed target of 0.2, 0.3 and 0.4 rad).

**Figure 3.**
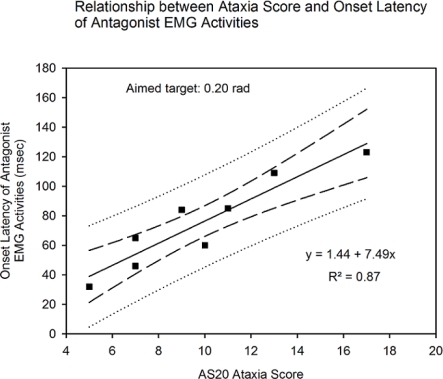
Relationship between the ataxia rating score and the onset latency of the antagonist EMG activities for fast pointing movements (FPM) (aimed target: 0.2 rad; basal mechanical state of the hand). Long dash: 95% confidence intervals; dotted lines: 95% prediction intervals.

The AS20 ataxia score was also linearly correlated with the onset latency of the antagonist EMG activity. Figure 3 illustrates an example for the aimed target of 0.2 rad (p < 0.001). Similar findings were found for an aimed amplitude of 0.3 and 0.4 rad.

**Figure 4.**
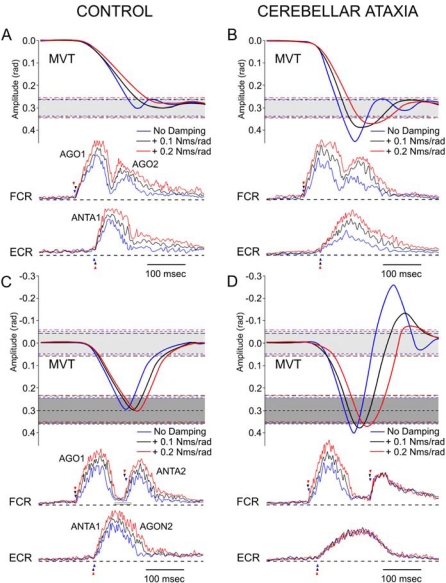
Movement and EMG bursts in a control subject and in a cerebellar patient (aimed target: 0.3 rad). Top panels (A,B): average of fast pointing movements (FPM). Bottom panels (C,D) refer to fast reversal movements (FRM). Blue line: no damping, black line: + 0.1 N·m·s/rad, red line: + 0.2 N·m·s/rad. Grey areas: 99% confidence interval of control values of movement amplitudes in the basal mechanical state; dotted lines in black and red: 99% confidence interval of control values during addition of 0.1 N·m·s/rad and 0.2 N·m·s/rad, respectively.

Figure 4 illustrates an example of the movement and the associated EMG activities in a control subject and a cerebellar patient. In the control subject, the pointing movements performed without damping are associated with a triphasic pattern of EMG activity (Figure 4A): a first burst in the FCR muscle (AGO1) is followed by a burst in the ECR muscle (ANTA1). This second burst is followed by another burst in the agonist muscle (AGO2). The control subject is able to increase the intensity of the 3 bursts of EMG activities when damping is applied. Movement remains accurate in the 3 experimental conditions (with a stabilizing effect induced by damping, as seen by damped oscillations around the aimed target). In the patient, pointing movements are characterized by an overshoot (the rate of rise in the antagonist EMG activity is depressed, [10]), which is decreased after addition of artificial damping (Figure 4B). The patient is able to tune the intensities of both the agonist and the antagonist EMG activities facing the artificial damping. In the healthy subject, reversal movements are accurate in the 3 mechanical conditions (Figure 4C). Reversal movements are associated with a burst of activity (AGO1) in the FCR muscle, followed by a burst of activity (composed of 2 fused components ANTA1/AGON2) in the ECR muscle (not only to provide the braking torque -ANTA1-, but also the second launching torque in order to return to the initial position: AGON2), followed by another burst of activity (ANTA2) in the FCR muscle (to provide the braking torque before the return to the initial position). The two bursts in the FCR muscle are clearly separated as previously reported [3]. Addition of damping is associated with an increase in the intensity of the two EMG bursts in the FCR muscle and the burst in the ECR muscle. For the patient (Figure 4D), movement performed in the basal condition is hypermetric during the first phase (towards the first target) and the second phase of movement (during the return to the starting position). Hypermetria is decreased with addition of damping. The patient increases the intensity of the first agonist burst (AGO1) in the FCR muscle, but cannot adapt the intensity of activity in the ECR muscle and cannot scale the magnitude of the second burst (ANTA2) in the FCR muscle. Data illustrated here for the control subject were representative of all recorded movements in the control group. For patients, the inability to tune appropriately the intensity of activity in the ECR muscle and the second burst in the FCR muscle during FRM was also representative.

**Figure 5.**
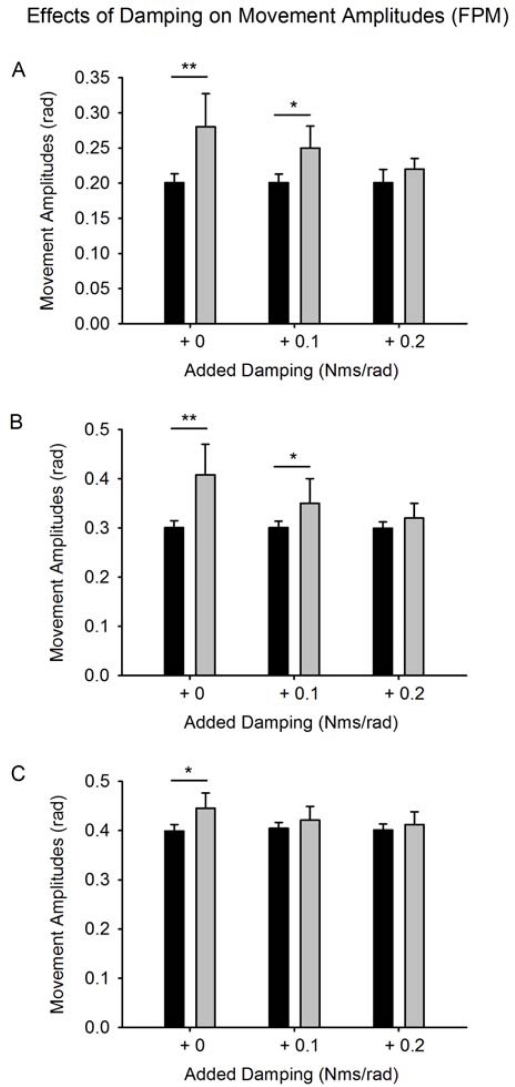
Effects of damping on movement amplitudes for fast pointing movements (FPM). Aimed target: 0.2 rad, 0.3 rad and 0.4 rad, respectively in A, B and C. Values are mean ±SD. Black columns: control subjects, grey columns: cerebellar patients. *: p < 0.05; **: p < 0.01.

Figure 5 shows the effects of damping on movement amplitudes for FPM. Damping does not modify the amplitudes of movement in control subjects, but damping attenuates significantly the hypermetria for the 3 aimed amplitudes in patients (group by damping effect: p = 0.002).

**Figure 6.**
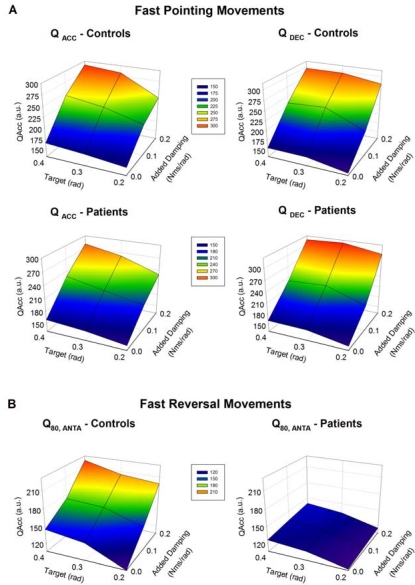
A: mesh plots illustrating the Q_ACC_ and Q_DEC_ in control subjects and patients for fast pointing movements (FPM). Mean values are shown for the 3 aimed targets and the 3 mechanical conditions. The scaling of the intensities of both agonist and antagonist EMG activities as a function of the damping and amplitudes of motion is preserved both in control subjects and in cerebellar patients. B: for fast reversal movements (FRM), control subjects scale appropriately the intensity of the activity of the extensor carpi radialis (ECR) muscle when damping is added and when the aimed amplitude is larger. By contrast, there is a failure of cerebellar patients to adapt the EMG activity as compared to controls.

Figure 6A illustrates the effects of damping on Q_ACC_ and Q_DEC_ in controls and in patients for FPM. Damping is associated with an increase in both Q_ACC_ and Q_DEC_ in the 2 groups. The increase is similar, both for the Q_ACC_ (inter-group effect: p = 0.33) and the Q_DEC_ (inter-group effect: p = 0.18). By contrast, control subjects scale appropriately the Q_80,ANTA_ during FPM, whereas patients show an inability to tune the intensity of the ECR muscle (Figure 6B; inter-group effect: p = 0.009).

**Figure 7.**
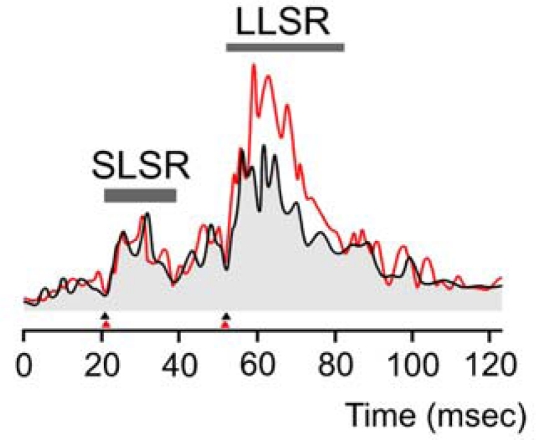
Representative EMG activities for the flexor carpi radialis muscle (FCR) during rapid stretches of the wrist (extensions) in a control subject (black trace) and a cerebellar patient (red trace). The intensities of the short-latency stretch response (SLSR) are similar. The intensity of the long-latency stretch response (LLSR) is higher in the patient. Each trace corresponds to an average of 45 trials. Stretch responses are calibrated in arbitrary units. Arrowheads at the bottom of the traces indicate the onset latencies of SLSR and LLSR.

Figure 7 illustrates an example of EMG activities in the FCR muscle in response to rapid stretches (extension of the wrist) for a control subject and a cerebellar patient. Intensities of SLSR were similar in the 2 groups, but magnitudes of LLSR were enhanced in patients. The ratios LLSR/SLSR were significantly higher in patients (mean ± SD: 2.29 ± 0.47) as compared to controls (1.48 ± 0.17; inter-group difference: p = 0.003)

**Figure 8.**
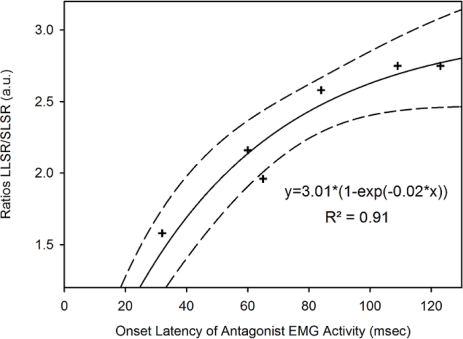
Fitting of the relationship between onset latency of antagonist EMG activity and the ratios LLSR/SLSR during fast pointing movements (FPM) towards an aimed target of 0.2 rad in cerebellar patients. Medium dash: 95% confidence band.

Figure 8 shows the relationship between the onset latency of the antagonist muscle for FPM (aimed target: 0.2 rad) and the ratios LLSR/SLSR in the group of patients with a predominant cerebellar cortical atrophy. The fitting with exponential rise to maximum showed a significant correlation between onset latency of antagonist EMG activity and the ratios LLSR/SLSR (p = 0.003). Similar observations were made for the aimed target of 0.3 rad and 0.4 rad.

## 4. Discussion

We report on a novel myohaptic device and an application in cerebellar patients. We show for the first time that the myohaptic technology allows the exploration of the motor commands in cerebellar patients by extracting an electrophysiological signature in terms of inability to adapt to artificial damping. Moreover, our results confirm that the instrument allows the investigation of the intensities of LLSR in cerebellar patients. Previous studies have demonstrated that cerebellar cortical lesions enhance these responses by disinhibiting the activities of cerebellar nuclei, because of the damage to cerebellar cortex [6,17]. We did not address the issue of the effects of ageing in this study. It is well known that motor unit characteristics are impaired by the ageing process and that the motoneurons which are lost are predominantly the larger motoneurons with higher recruitment thresholds. However, this alteration cannot be studied with our EMG recordings and the assessment of the subtle changes occurring in the descending motor drive require other techniques. Performances for wrist motion are highly dependent on the general level of physical activity. We did not address the relationship between body mass index (BMI) and performance in our test. Surface EMG activities are highly influenced by level of fat under the skin, which varies amongst subjects [7–9], hence the interest of calibration techniques to compare EMG activities amongst different subjects.

Other applications include the evaluation of upper limb tremor in various damping conditions and rehabilitation of wrist disorders [12]. The advantages of the instrument are its robustness, the comfort during testing and the reliability of the procedures [12]. By contrast, although it can be moved easily to a bedside, it has obvious disadvantages for evaluation of multi-joint free motion as compared to small and light sensors such as gyroscopes and accelerometers. These sensors have the valuable feature of being unobtrusive [19,20]. They can be integrated into wearable devices, a field which is expanding quickly in bioengineering.

In terms of motor strategy, a major rule used by the CNS is to increase the intensity of the agonist EMG activity for movements requiring a greater impulse [3,7–10]. Our current hypothesis about programming of motor commands related to “ballistic” movements (movements performed as fast as possible) is that the full set of muscle discharges is triggered in a feed-forward manner, using an error-feedback learning [21–23]. In other words, once the program is launched, it cannot be changed during movement execution given the speed of motion. In more complex movements, the motor plan consists of a superimposition of elemental defined components [24,25]. The elemental components need to be selected and to be superimposed sequentially. One way to characterize the problem the CNS faces in *planning* a movement is that it must *anticipate* how to generate these appropriate patterns of muscle activation [3], in agreement with the prevailing idea that the cerebellum stores internal models of the motor system and that Purkinje cells predict the kinematics of arm movements [22–26]. So far, the structures of the CNS responsible for the *sequential superimposition* of the individual components underlying motor commands have not been identified. We show for the first time with the myohaptic technology that the cerebellar circuitry plays a key-role in this fundamental process underlying human motor control. Complexity of brain circuitry contributes to the challenging goal of extracting signatures related to motor action [27]. Since the ability of the CNS to adapt to the variations of passive torques occurring during motion permits the control of angular position parameters [28], the distorted pattern reported here might be an elemental and so far not recognized defect underlying cerebellar dysmetria.

When we asked our patients whether addition of damping improved or decreased the accuracy, they all said that it was more difficult to perform FRM with damping, while the accuracy was improved with damping the FPM. This highlights a difference between the adaptation to inertia and the adaptation to damping when cerebellar patients perform pointing movements, since cerebellar hypermetria is larger when the mass is artificially increased [7]. It is well know from clinical practice that the faster the movement, the greater cerebellar dysmetria. Since faster movements are associated with greater damping [13], the inability of cerebellar patients to adapt to damping might explain this observation. We thus suggest that 5 core deficits underlie human cerebellar dysmetria: a delayed onset latency of the antagonist EMG activity, a prolongation of the first agonist EMG activity, a reduced rate of rise of the antagonist EMG activity, an inability to adapt to inertia and an abnormal compensation to damping [6].

## 5. Conclusions

We describe here a new instrument which enabled the unraveling of a new muscle activation pattern during single-joint sequential movements. Our patients were unable to tune the intensity of specific components of the pattern of EMG activity, while the modulation of other components was preserved. In particular, we show that a given muscle can exhibit a normal behavior facing mechanical damping during the first part of a motor sequence, but cannot adapt appropriately for the next part. Rehabilitation strategies in patients with cerebellar disorders should take into account the differences in the motor strategies underlying pointing movements and reversal movements in opposite directions in cerebellar disorders. This is not the case currently. We hypothesize that our patient might not get access to some specific sections of the motor code under the experimental condition of artificial damping. The code appears to be executed adequately in the first part of a complex movement, but its execution is aberrant in the second part. Studies under artificial damping reveal that the estimations of the motoneuronal discharges are wrong. The myohaptic technology opens new perspectives to understand the pathogenesis of motion deficits in neurological patients. Given the rapid development of new therapies for neurological disorders which were considered as not curable a decade ago [29–31], there is a growing need for clinically-oriented standardized tools for the monitoring of neurological patients with movement disorders.
